# Neuronal THY1 Signaling Maintains Astrocytes in a Quiescent State

**DOI:** 10.1002/glia.70083

**Published:** 2025-09-11

**Authors:** Juliane Loui, Ute Krügel, Ulrike Winkler, Anja Reinert, Dorit John, Johannes Hirrlinger, Anja Saalbach

**Affiliations:** ^1^ Department of Allergology, Venerology and Dermatology Leipzig University Leipzig Germany; ^2^ Carl Ludwig Institute for Physiology, Leipzig University Leipzig Germany; ^3^ Rudolf Boehm Institute of Pharmacology and Toxicology, Leipzig University Leipzig Germany; ^4^ Medical Department II—Division of Oncology, Gastroenterology, Hepatology and Pneumology University Clinical Center Leipzig Leipzig Germany; ^5^ Department of Neurogenetics Max Planck Institute for Multidisciplinary Sciences Göttingen Germany

**Keywords:** astrocyte, neuron, THY1

## Abstract

THY1 is a cell surface protein of mature neurons. Although the *Thy1* promoter is widely used as a neuron‐specific promoter for transgenic expression, the role of the endogenous THY1 protein in the brain remains largely unknown. As THY1 receptors are expressed on astrocytes, THY1 may mediate signaling between both cell types. We therefore investigated the role of THY1 signaling in neuron‐astrocyte communication using a full as well as a neuron‐specific *Thy1*‐knockout mouse model. Compared to wild‐type mice, aged individuals of both strains exhibited an increased expression of a subset of astrocyte activation‐associated genes, such as glial fibrillary acidic protein (*Gfap*), vimentin (*Vim*), and tenascin C (*Tnc*), whereas others appeared unaffected. Importantly, a cortical injury caused a permanent astrocytic activation in mice with neuronal *Thy1* deletion, reflected by persistent high GFAP expression. The THY1‐associated modulation of gene expression was confirmed in primary astrocytes cultured with or without recombinant THY1. Moreover, functional assays indicate that THY1 inhibits astrocyte proliferation while promoting apoptosis. Interaction of neuronal THY1 with ITGB1 on astrocytes was identified to be responsible for the THY1‐mediated control of astrocyte activation. These data strongly suggest that THY1‐bearing neurons keep astrocytes in a quiescent state. Consequently, a depletion of THY1 supports the development of a partially activated astrocyte phenotype characterized by increased expression of intermediate filaments, increased proliferative capacity, and reduced cell death. Our findings demonstrate that neuronal THY1 is a still unrecognized novel regulator in the communication between astrocytes and neurons involved in the maintenance and restoration of tissue homeostasis in the brain.

## Graphical abstract

- THY1‐bearing neurons keep astrocytes in a quiescent state via the interaction with ITGB1.
- Depletion of THY1 supports the development of a partially activated astrocyte phenotype.
- Neuronal THY1 is involved in the maintenance and restoration of tissue homeostasis in the brain.

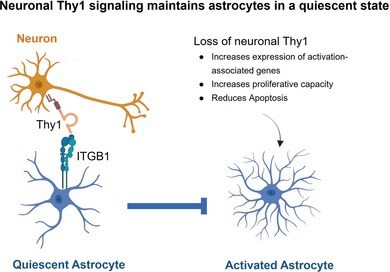

## 1 Introduction

Astrocytes are the most numerous glial cell type in the central nervous system (CNS). With perisynaptic astrocytic processes, they are in close and highly dynamic contact with neuronal synapses (Hirrlinger et al. 2004; Haber et al. 2006; Nishida and Okabe 2007; Theodosis et al. 2008; Perea et al. 2009; Henneberger et al. 2020). A single protoplasmic astrocyte in the human brain contacts up to 2 million neuronal synapses within its spatial domain (Pekny and Pekna 2014). Many signaling molecules have been identified that signal from neurons to astrocytes and vice versa, among them neuro‐ and gliotransmitters, cholesterol (Mauch et al. 2001), or secreted proteins like neurocan (Irala et al. 2024), thrombospondin‐4 (Christopherson et al. 2005), hevin, and sparc (Kucukdereli et al. 2011). Furthermore, a bidirectional signaling between neurons and astrocytes via cell adhesion molecules has been characterized, for example, via ephrinA3‐EphA4, ephrinB1‐EphB, neuroligin‐neurexin, g‐protocadherin, NrCam, and cadherins (Murai et al. 2003; Stogsdill et al. 2017; Nguyen et al. 2020; Pancho et al. 2020; Takano et al. 2020; Tan and Eroglu 2021; Tan et al. 2023). Such signals contribute to synaptogenesis, maturation of synapses, regulation of the excitation/inhibition balance, and development of astrocytes' morphology (Tan and Eroglu 2021). Furthermore, astrocytes recognize neurotransmitters via receptors, ensure potassium ion homeostasis, regulate extracellular pH, release signaling molecules such as ATP and d‐serine, and take up neurotransmitters like glutamate, glycine, and GABA, thereby crucially contributing to the control of neurotransmission (Oliet et al. 2001; Kimelberg 2010; Savtchouk and Volterra 2018; Verkhratsky and Nedergaard 2018; Santello et al. 2019; Hasel and Liddelow 2021). Astrocytes are embedded in neuron–glia assemblies in which they modulate network activity and finally the behavior of the organism (Akther and Hirase 2022; Bohmbach et al. 2023; Hirrlinger and Nimmerjahn 2022; Lawal et al. 2022; Oliveira and Araque 2022; Bonvento and Bolaños 2021; Mächler et al. 2016; Zuend et al. 2020).

Astrocytes are a heterogeneous cell population allowing each individual astrocyte to adapt to the local requirements (Khakh and Sofroniew 2015; Holt et al. 2019; Khakh and Deneen 2019; Escartin et al. 2021; Hasel and Liddelow 2021). During injury or inflammation, functional “quiescent” astrocytes are transformed into an activated state (Escartin et al. 2021; Pekny and Pekna 2014). This astrocyte activation has long been considered a uniform response to tissue damage (Burda et al. 2022). However, recent studies provided evidence that “reactive” astrocytes adopt remarkably heterogeneous states in different conditions and locations, which differ in the expression of activation‐related genes and in functions like regeneration, proliferation, and differentiation (Sofroniew 2009; Escartin et al. 2021). Numerous genes are predicted to be associated with the response of astrocytes in brain pathologies. However, in three experimental models of various CNS disorders, only 2.6% of differentially expressed genes were shared by all three disorders, demonstrating the contextually heterogeneous response of astrocytes (Burda et al. 2022). Therefore, there is an increasing interest in understanding the control of astrocyte activation under specific conditions.

One so far poorly understood signal in the bi‐directional communication between neurons and astrocytes might be THY1 (thymus cell antigen 1, theta, CD90), a highly conserved glycosylphosphatidylinositol‐anchored cell surface protein. It is expressed on mature neurons, activated microvascular endothelial cells, fibroblasts, and mesenchymal stem cells. In addition, in mice but not in humans, THY1 was found on T lymphocytes (Haeryfar and Hoskin 2004; Hu et al. 2022). With an abundance of 2%–7% in the striatum, hippocampus, neocortex, cerebellum, and spinal cord, it represents one of the most abundant cell‐surface glycoproteins in the nervous system (Barlow et al. 2002). THY1 expression is mostly suppressed in brain regions where active axonal growth takes place (Chen et al. 2005).

Mice lacking THY1 do not differ from their wild‐type (WT) littermates in breeding, overall behavior, health, neuroanatomy, cellular architecture, axonal guidance, pathway formation, and basic properties of synaptic neurotransmission (Nosten‐Bertrand et al. 1996; Barlow et al. 2002).

Detailed morphological analysis demonstrates normal cellular organization, normal anatomical features of the corticospinal and thalamocortical pathways, and basic neurophysiological properties of thalamocortical synaptic transmission, which were quantitatively indistinguishable from WT mice (Barlow et al. 2002). Moreover, no differences were observed in the stratified arrangement of terminal fields in the hippocampus, and the distribution of GABAergic inhibitory interneurons and the number and affinity of NMDA‐type glutamate receptors do not differ from WT mice (Nosten‐Bertrand et al. 1996). Mice lacking THY1 exhibit a deficit in long‐term potentiation (LTP) specifically in the dentate gyrus but not in the CA1 region of the hippocampus, which might be due to subtle alteration of GABAergic inhibitory transmission (Nosten‐Bertrand et al. 1996). Furthermore, distinct defects in the social transmission of food preference were observed (Nosten‐Bertrand et al. 1996; Mayeux‐Portas et al. 2000; Chen et al. 2005). Overall, the observed impairments induced by *Thy1* deletion in vivo are surprisingly subtle, given the high abundance of THY1 on the surface of neurons, amounting to 2.5%–7.5% of all surface proteins on axons (Barlow et al. 2002).

Various receptors have been described for THY1 in mouse and human. So far, for example, integrin β1 (ITGB1), β2 (ITGB2), β3 (ITGB3), β5 (ITGB5) as well as ADGRE5 (Adhesion G Protein‐Coupled Receptor E5, CD97) and syndecan‐4 (Syn4) were described to recognize THY1 (Avalos et al. 2002, 2009; Wetzel et al. 2004; Saalbach et al. 2005; Hermosilla et al. 2008; Zhou et al. 2010; Wandel et al. 2012). In contrast to other cell types, the function of neuronal THY1 is poorly understood. On microvascular endothelial cells, THY1 mediates the adhesion and transmigration of myeloid cells via Mac‐1 (CD11b/CD18) and CD97 (Wetzel et al. 2004; Wandel et al. 2012). Expressed on mesenchymal stem cells, THY1 balances osteogenesis and adipogenesis and thus promotes bone formation while inhibiting adipogenesis and obesity (Picke et al. 2018). Furthermore, on fibroblasts, THY1 is essential for the control of fibrogenesis, including proliferation, apoptosis, responsiveness to different cytokines, cell adhesion, migration, ECM deposition, and myofibroblast differentiation (Hu et al. 2022). Data from these cell types show that THY1 seems to be a key player in controlling the balance between proliferation and differentiation, which is essential for maintaining and restoring tissue homeostasis. This raises the question of specific effects that neuronal THY1 may mediate in astrocytes. Here, we hypothesized that THY1 contributes to adhesion‐mediated signaling from neurons to astrocytes. Using a full *Thy1*‐knockout mouse model as well as a mouse line with neuron‐specific deletion of *Thy1*, we demonstrate that neuronal THY1 is a still unrecognized novel regulator in the communication between astrocytes and neurons controlling the activation state of astrocytes and, consequently, the maintenance and restoration of tissue homeostasis in the brain.

## 2 Materials and Methods

### 2.1 Animal Studies

*Thy1*‐deficient mice (Thy1‐KO, C57BL/6N‐Thy1^−/−^) were kindly provided as a gift from Dr. R. Morris (King's College London, UK; Nosten‐Bertrand et al. 1996). A neuron‐specific knockout mouse (nexThy1‐KO) was generated by crossing a mouse line with a loxP‐flanked *Thy1* allele (Thy1^fl/fl^; Schmidt et al. 2016) to the Nex‐Cre driver line, which induces Cre‐dependent DNA recombination specifically in neurons (Neurod6^tm1(cre)Kan^; Goebbels et al. 2006). In Nex‐Cre driver mice, the vast majority of neurons in the cortex, the brain area analyzed in this study, are targeted by DNA recombination. Mice were accommodated in individually ventilated cages within a specific pathogen‐free environment, following a 12‐h light/12‐h dark cycle, with unrestricted access to food and water. All animal experiments were performed in accordance with welfare guidelines outlined by the European Communities Council Directive (2010/63/EU) and the German Protection of Animals Act as well as institutional guidelines and received approval from the animal welfare office of the University Medical Center, Leipzig, as well as from the local authorities (Committee on Animal Welfare of Saxony at the Landesdirektion Leipzig), with registration numbers TVV39/23, TVV26/19, and T05/21‐MEZ.

### 2.2 Cortical Injury Model

NexThy1‐KO and control mice (mice with loxP‐flanked *Thy1* allele [Thy1^fl/fl^]), analgesized with metamizole and anesthetized with isoflurane, were fixed in a stereotaxic frame on a heating pad (TSE, Germany). After opening the skull, a sterile stainless‐steel cannula (30G; OD = 0.3 mm) was inserted into the brain at 1.4 mm posterior and 0.7 mm lateral to the bregma to a depth of 4.3 mm from the skull surface. The cannula was left there for 2 min. After removing the cannula, the skin edges were sealed with tissue adhesive (Surgibond; SMI AG, Belgium). After surgery, the animals received metamizole for 24 h with drinking water. Since THY1 is mainly deleted in cortical neurons in this mouse strain, the impact of *Thy1* deletion on astrocyte action upon wounding was analyzed specifically in the cortical region.

### 2.3 Induction of Inflammation

Adult C57BL/6N mice were anesthetized. The animals were shaved on the dorsal side and sensitized by epicutaneous application of 100 μL 5% TNCB (2‐chloro‐1,3,5‐trinitrobenzene; Sigma) in acetone:olive oil (4:1). After 24 h, the cortex was isolated.

### 2.4 Isolation of Astrocytes by Magnetic Cell Sorting

Magnetic sorting of astrocytes was performed using the Adult Brain Dissociation Kit (Miltenyi Biotec, Bergisch Gladbach, Germany), and subsequently the Anti‐ACSA‐2 MicroBead Kit as described in the manufacturer's instructions. Anti‐ACSA‐2 antibody reacts specifically with glycosylated surface molecules on mouse astrocytes at all developmental stages, while it does not bind to any non‐astroglial cells (Kantzer et al. 2017). In detail, the brains of adult mice were prepared and washed in ice‐cold phosphate‐buffered saline (PBS). The cortices were dissected under sterile conditions and enzymatically dissociated, followed by mechanical dissociation with a gentleMACS Octo Dissociator with heaters (Miltenyi Biotec). Myelin and debris were removed via density gradient centrifugation using Debris Removal Solution. Red blood cells were eliminated with Red Blood Cell Removal Solution. Astrocytes were magnetically labeled with Anti‐ACSA‐2 MicroBeads and separated on MS Columns and Separators (Miltenyi Biotec). Purity of the astrocyte fraction was assessed via flow cytometry. RNA from isolated cells was extracted by means of a ReliaPrep RNA Miniprep system (Promega, Walldorf, Germany).

### 2.5 P0/1 Astrocyte Isolation and Culture

Astroglia‐rich primary cultures derived from the brains of neonatal C57BL/6N mice were prepared and maintained as described elsewhere (Winkler et al. 2017). Briefly, cerebral cortices from postnatal day 0 or 1 mouse pups were filtered through nylon meshes with pore sizes of 210 and 132 μm. Prior to cell seeding, 24‐well plates were coated overnight with 0.1% gelatin. Subsequently, a total of 3 × 10^5^ viable cells per well were seeded in 24‐well plates in 0.5 mL Dulbecco's modified Eagle medium (DMEM) containing 25 mM glucose, 4 mM l‐glutamine, 1 mM sodium pyruvate, 10% fetal calf serum (FCS), 20 units/mL penicillin G, and 20 μg/mL streptomycin sulfate. The cells were cultured in a humidified atmosphere of 5% CO_2_ and 95% air at 37°C. One week after seeding, the medium was replaced with DMEM containing 5 mM glucose, 4 mM l‐glutamine, 1 mM sodium pyruvate, 10% FCS, 20 units/mL penicillin G, and 20 μg/mL streptomycin sulfate. Medium was then exchanged twice a week thereafter. The cells were harvested after 7–14 days.

### 2.6 Functional Assays

96‐well High Binding ELISA plates (Sarstedt, Nümbrecht, Germany) were coated with 5 μg/mL recombinant THY1 (rTHY1, RLD) fused to Fc protein, mutated rTHY1 (RLE) or Fc protein as control (R&D Systems, Minneapolis, MN, USA) in coating buffer containing 0.2 M Na_2_CO_3_ and 0.2 M NaHCO_3_ (pH 9.5) overnight at 4°C. After culturing astrocytes for 7–14 days as described above, cells were detached by TrypLE Express (Gibco, Dreieich, Germany) and 4 × 10^4^ cells were seeded on pre‐coated wells. Following a 2‐day cultivation on rTHY1 or Fc, functional assays were performed. In experiments with soluble THY1, 5 μg/mL recombinant THY1 was added in soluble form (sTHY1) to the culture medium, and cells were also cultured for 2 days.

*Cell growth* analysis was performed by a 2,3‐bis‐(2‐methoxy‐4‐nitro‐5‐sulfophenyl)‐2H‐tetrazolium‐5‐carboxanilide salt (XTT) assay (Roche, Mannheim, Germany). The absorbance at 490 nm was measured 2 h after the addition of XTT at a microplate reader (BioTek Instruments, Winooski, Vermont, US). As indicated, 10 μM Y27632 (Miltenyi Biotech), a selective small molecule inhibitor of Rho‐associated kinase (ROCK), was added.

*Cell proliferation* was quantified by BrdU incorporation during DNA synthesis after 48 h with the Cell Proliferation ELISA assay (Roche, Mannheim, Germany) according to the manufacturer's protocol. Measurement of absorbance at 490 nm was performed at a microplate reader (BioTek Instruments, Winooski, VT, USA).

*Apoptosis* was detected by measuring the activity of caspase 3/7 using Caspase‐Glo 3/7 Assay (Promega, Madison, USA) according to the manufacturer's protocol. The luminescence signal was detected after 120 min by a microplate reader (BioTek Instruments, Winooski, VT, USA). To exclude differences in caspase 3/7 activity due to different proliferation rates, the luminescent signal was expressed per 10,000 cells.

### 2.7 Transfection

TU2449 cells (Pohl et al. 1999) were transfected with 60 nM *Itgb1* siRNA (Santa Cruz, CA, USA), 25 nM *Itgb5* (Horizon, Cambridge, UK), or 25 nM scrambled siRNA (Horizon) mixed with Lipofectamine 3000 (Thermo Fisher Scientific) and Opti‐MEM (Thermo Fisher Scientific) for 72 h. Transfection efficiency was assessed by flow cytometry. After harvesting, 6 × 10^3^ cells were seeded per well into 96‐well plates pre‐coated with either recombinant THY1 fused to Fc protein or Fc protein as a control for cell growth analysis using the XTT‐Assay after 2 days.

### 2.8 Brain Slice Culture

Four‐week‐old C57BL/6N mice were sacrificed, the brain was immediately isolated, and 300‐μm‐thick whole‐brain sections were cut and collected in sterile medium. Brain slices were cultured in DMEM with 25 mM glucose, 10% FCS, 20 units/mL penicillin G, 20 μg/mL streptomycin sulfate, and insulin‐transferrin‐selenium supplement (Gibco). After 3 days, the medium was replaced, and slice cultures were stimulated with 10 ng/mL TNF‐α and 10 ng/mL IL‐1β (Miltenyi Biotech) for 24 h.

### 2.9 Gene Expression Analysis

Total RNA was isolated from mouse cortex or cells by means of the ReliaPrep RNA Miniprep system (Promega) according to the manufacturer's protocol. Total RNA (0.5 μg) was used for cDNA synthesis using LunaScript RT Supermix (New England Biolabs, Ipswich, MA, USA) according to the manufacturer's instructions. Real‐time qPCR was performed with LunaUniversal qPCR Mastermix (New England Biolabs) following the manufacturer's instructions. Quantitative gene expression was calculated from the standard curve of the cloned cDNA and normalized to the reference gene *Rplp0*. The sequences of primers used are listed in Table S1.

### 2.10 Tissue Staining

#### 2.10.1 Staining for GFAP, NeuN, and THY1

Brains were isolated, rinsed with ice‐cold PBS, embedded in Tissue freezing medium (Leica Biosystems), and subsequently frozen at −80°C until further processing. Tissue sections were generated by cryotome at −20°C. Brain sections (13 μm) were fixed with acetone for 10 min, incubated overnight with primary antibody (anti‐GFAP, anti‐NeuN, and anti‐THY1) in PBS/3% BSA/0.1% saponin, and then washed in PBS/0.05% Tween 20. The details on the antibodies used are listed in Table S2. Depending on the primary antibody, Alexa Fluor 647 goat anti‐rat or goat anti‐rabbit IgG (Invitrogen, MA, USA) antibodies were added for 1 h. Nuclei were stained with DAPI. Sections were imaged with a fluorescence microscope BZ‐X810 with a monochromatic CCD camera (Keyence, Neu‐Isenburg, Germany). Images were taken at 4× or 10× magnification, and quantification was done using the Hybrid Cell Count module of the BZ‐X800 Viewer software. The number of positive cells or positively stained areas within a defined region was determined.

#### 2.10.2 Staining for 3‐Phosphoglycerate Dehydrogenase (3PGDH) and Aquaporin‐4 (AQP4)

Mice were transcardially perfused with 4% paraformaldehyde (PFA), their brains postfixed in PFA for 24 h and sliced with a vibratome in 40‐μm‐thick sections. Slices were permeabilized in 0.4% Triton X‐100/PBS for 30 min and blocked with 4% FCS in 0.2% Triton X‐100/PBS for 30 min. Sections were incubated overnight at 4°C in primary antibody solution (1% FCS, 0.05% Triton X‐100/PBS) containing anti‐3PGDH and anti‐AQP4 antibodies. After washing, sections were incubated with secondary antibodies, goat anti‐guinea pig Cy3 and goat anti‐rabbit Cy5 in 1.5% FCS/PBS for 2 h at room temperature. Sections were mounted in ImmunoSelect Antifading Mounting Medium (Dianova) and examined using a Zeiss Airyscan 880 confocal laser scanning microscope with a 10×, 20×, and 63× objective. Image analysis and cell counting were performed using the ZEN (Zeiss) and QuPath software.

### 2.11 FACS

The purity and phenotype of the isolated cells were determined by flow cytometry and the respective software (BD FACSLyric flow cytometer, BD FACSuite RUOv1.3 BD Bioscience, both Franklin Lakes, NJ, USA) with the following antibodies: Anti‐ACSA‐2, anti‐O4, anti‐CD11b, anti‐CD29, anti‐CD61, and anti‐ITGB5. Dead cells were excluded with Zombie NIR (BioLegend, San Diego, USA). Antibody details are listed in Table S2.

### 2.12 Western Blot

Cortex was isolated and homogenized in 100 μL ProcartaPlex Cell Lysis Buffer (Thermo Fisher Scientific, Karlsruhe, Germany) containing 1 mM PMSF (Miltenyi) using 5 mm Stainless Steel Beads (Qiagen, Hilden, Germany) and TissueLyser LT (Qiagen). Lysates were centrifuged for 10 min at 16,000 × *g*, at 4°C. Proteins were separated by 8%–16% SDS PAGE (Bio‐Rad, Munich, Germany) and blotted onto PVDF membrane. Proteins were detected with anti‐THY1 (BD, Franklin Lakes, NJ, USA), anti‐RPL26 (Sigma‐Aldrich, Taufenkirchen, Germany), or anti‐GFAP antibodies (Cell Signaling Technology, Leiden, the Netherlands). After overnight incubation and washing steps, anti‐rabbit or anti‐rat IRDye680 labeled antibodies (Invitrogen/TFS, Waltham, MA, USA) were added, followed by washing and detection at LI‐COR Odyssey Fc (LI‐COR Inc., Lincoln, NE, USA). Signals of investigated proteins were normalized to the reference protein RPL26. Antibody details are listed in Table S2.

### 2.13 Statistical Analysis

Significance was analyzed with GraphPad Prism 10 (GraphPad Software, San Diego, USA). Normality was tested by the D'Agostino & Pearson Normality test or the Shapiro–Wilk test (*n* ≤ 4). Normally distributed data were analyzed using a two‐tailed Student's *t*‐test. Non‐normally distributed data were analyzed by the Mann–Whitney *U*‐test. A one‐way ANOVA test was used for statistical comparison of more than two groups. *p* values less than 0.05 were considered significant.

## 3 Results

### 3.1 THY1 Receptor Expression in Brain

**FIGURE 1:**
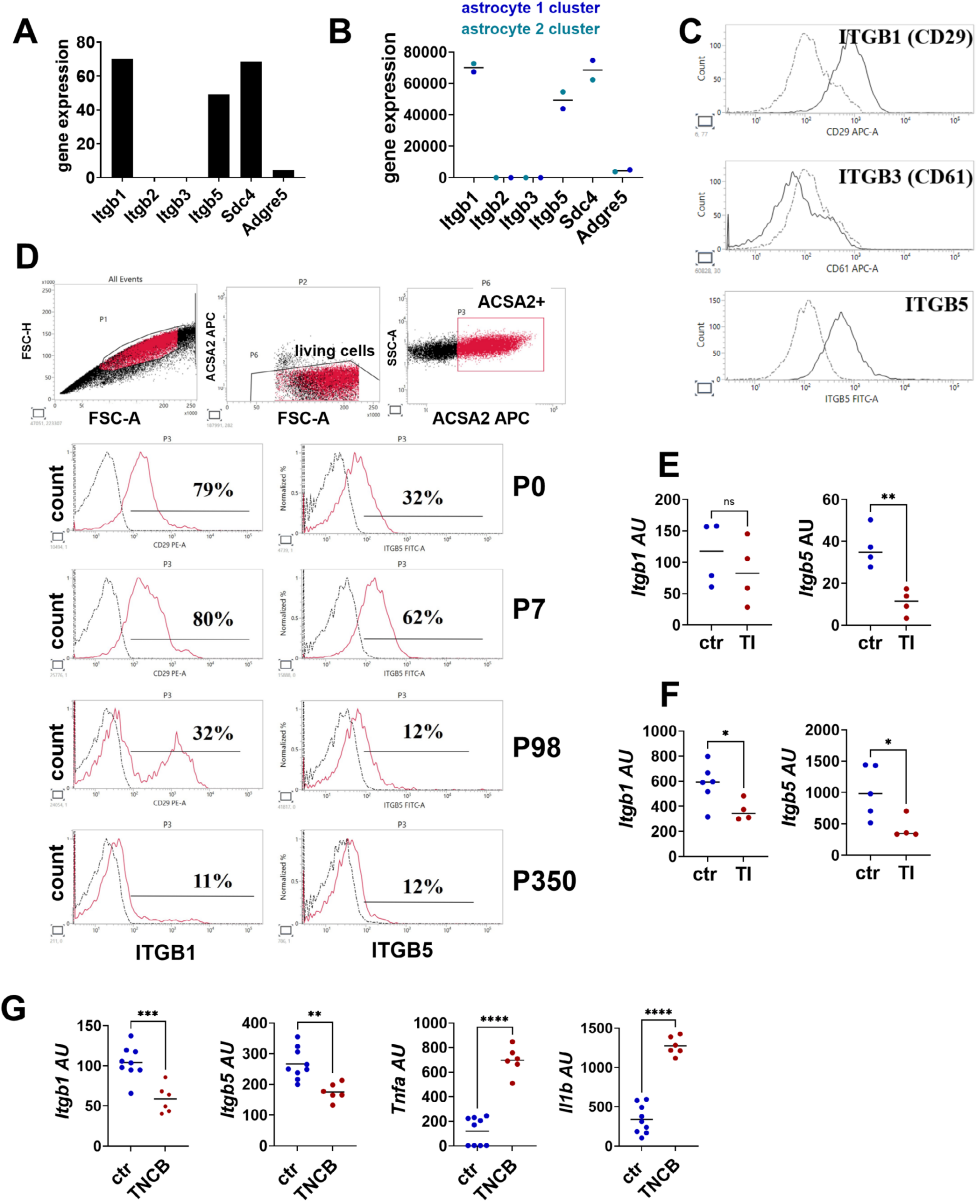
Expression of THY1‐receptors on astrocytes. (A and B) Expression of THY1 receptors detected in publicly accessible datasets. (A) Expression of THY1 receptors, integrin beta 1, 2, 3, and 5 (*Itgb*), syndecan‐4 (*Sdc4*), and adhesion G Protein‐Coupled Receptor E5 (*Adgre5*) in astrocytes (http://www.brainrnaseq.org/). (B) Single nuclei sequencing data from the cortex of adult mice (Allen Brain Atlas; https://celltypes.brain‐map.org/). Expression of THY1 receptors in two astrocyte cell clusters. (C) Flow cytometry analysis of ITGB1, ITGB3, and ITGB5 expression (black line) on in vitro cultured astrocytes from P0/P1 mice. ACSA‐2 staining was used to select astrocytes. Isotype control antibody (gray line) was used as control. One representative experiment of three is shown. (D) Flow cytometry analysis of integrin expression (red) on astrocytes from the cortex of newborn (P0), P7, adult (P98), and aged (P350) mice. ACSA‐2 staining was used to select astrocytes. Isotype control antibody (black) was used as control. One representative experiment of three is shown. (E) *Itgb1* and *Itgb5* gene expression in brain slice cultures with and without stimulation with TNF‐α/IL‐1β (TI) for 24 h. (F) *Itgb1* and *Itgb5* gene expression in primary astrocyte culture with and without stimulation with TNF‐α/IL‐1β (TI) for 24 h. (H) Gene expression of indicated genes in the cortex of untreated and trinitrobenzol‐treated (TNCB) mice. (E–G) Each point represents one mouse (*n* = 4–6). The black line represents the mean. *p < 0.05, **p < 0.01, ***p < 0.001, ****p < 0.0001, ns: not significant, Student's *t*‐test.

To verify THY1 receptor expression on astrocytes, a dataset of RNA sequencing data of isolated cell populations from the mouse cerebral cortex was re‐analyzed (http://www.brainrnaseq.org/) (Zhang et al. 2014). Among the already identified THY1 receptors, only *Itgb1*, *Itgb5*, and *Sdc4* showed detectable mRNA expression in astrocytes according to this database (Figure 1A). Single nuclei sequencing data from the cortex of adult mice (Allen Brain Atlas; https://celltypes.brain‐map.org/) confirm the expression of these three THY1 receptors on astrocytes (Figure 1B). To verify the presence of THY1 receptors on astrocytes, we analyzed the expression of ITGB1, ITGB3, and ITGB5 by flow cytometry. Astrocytes were isolated from P0/1 WT mice and cultured in vitro. Indeed, ITGB1 and ITGB5 could be detected on astrocytes identified by ACSA‐2 staining, whereas ITGB3 was not detectable on this cell type (Figure 1C). Finally, integrin expression was measured on astrocytes isolated from the brain at different developmental stages. A single‐cell suspension was obtained by enzymatic digestion of the cortex. Upon removal of cell debris and erythrocytes, integrin expression was measured by flow cytometry. Astrocytes were identified by ACSA‐2 staining. Consistent with the RNA data and results from in vitro cultured astrocytes, freshly isolated astrocytes expressed ITGB1 and ITGB5 (Figure 1D). Astrocytes from P0 and P7 mice display high expression of ITGB1 and ITGB5. Interestingly, the expression of both integrins declines with further development (Figure 1D). Moreover, integrin expression is downregulated under inflammatory conditions. Stimulation of brain slice cultures with TNF‐α/IL‐1β to imitate an inflammatory microenvironment results in a reduction of the gene expression of both integrins (Figure 1E). To validate that this effect is due to the regulation of integrin expression on astrocytes, primary astrocytes from P0/1 mice were stimulated with TNF‐α/IL‐1β. Indeed, both *Itgb5* and *Itgb1* expression were significantly inhibited by TNF‐α/IL‐1β (Figure 1F). Finally, the expression of both integrins was investigated under inflammatory conditions in vivo. Inflammation was induced by epicutaneous application of TNCB, resulting in upregulation of *Tnfa and Il1b* in the cortex (Figure 1G). Indeed, under inflammatory conditions when *Tnfa and Il1b* are upregulated, the expression of both integrins is downregulated (Figure 1G).

### 3.2 Loss of THY1 Affects Astrocyte Activation

The astrocytic expression of THY1 receptors suggests that THY1 mediates a direct neuron‐to‐astrocyte signaling. To resolve such an unknown function in neuron‐astrocyte communication, whole‐body *Thy1*‐deficient mice (Thy1‐KO) were used (Nosten‐Bertrand et al. 1996). As expected, while THY1 was readily detectable in control mice, THY1 was absent in Thy1‐KO mice, as shown by immunofluorescence staining (Figure S1A) and Western blot (Figure S1B). Staining of neurons by NeuN did not display any differences in the number of NeuN^+^ cells in the cortex of WT and Thy1‐KO mice (Figure S1C,D). Flow cytometry analysis of single cells from the cortex confirmed that the distribution of astrocytes, microglia, oligodendrocytes, or neurons is similar in control and Thy1‐KO mice (Figure S1E,F).

**FIGURE 2:**
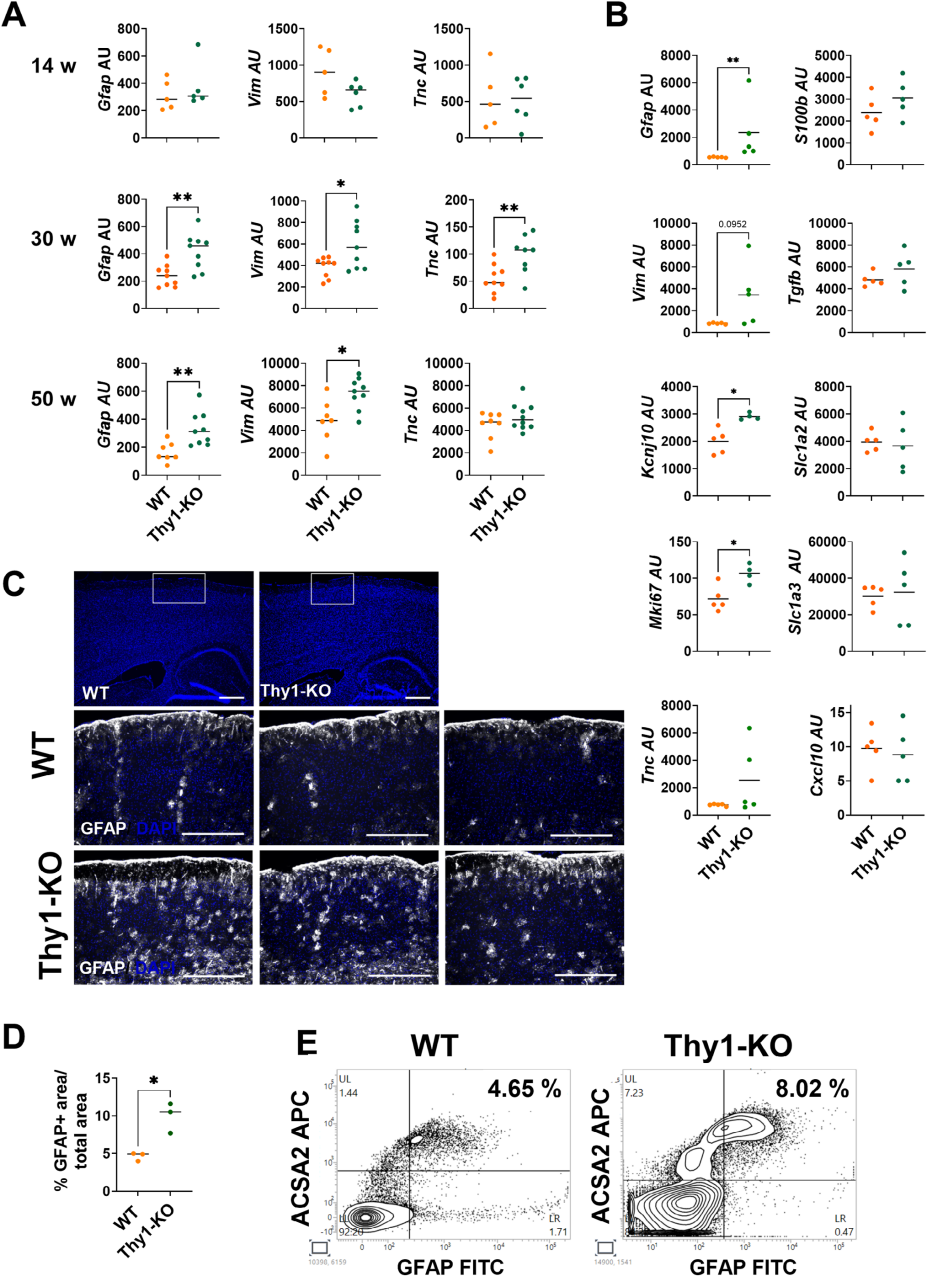
Deletion of *Thy1* induces a specifically activated phenotype in astrocytes. (A) Gene expression of *Gfap*, *Vim*, and *Tnc* in the cortex of young adult (12–14 weeks), aged (30–40 weeks), and old (50 weeks) wild‐type (WT) and Thy1‐KO mice was detected by RT‐qPCR. (B) Astrocytes were isolated from aged mice by magnetic cell separation. Indicated genes were detected by RT‐qPCR. (C and D) Detection of GFAP (white) in brain slices of aged mice by immunofluorescence staining. Nuclei were labeled with DAPI (blue). White boxes indicate area of higher magnification. Images of three different mice are shown. Scale bar: 500 μm. (D) Area of GFAP‐positive signal was evaluated. Percentage of GFAP+ area/total area is shown. (E) Flow cytometry analysis of the cortex of aged WT and Thy1‐KO mice. GFAP and ACSA‐2 expression were detected in WT and Thy1‐KO. One representative example of three is shown. (A, B, and D) Each point represents one mouse (*n* = 3–10). The black line represents the mean. *p < 0.05, **p < 0.01, Student's *t*‐test.

Next, we asked whether THY1 signaling is involved in the regulation of the astrocytic activation state. To include potential age‐associated changes of astrocyte activation, young adult (12–14 weeks), aged (30–40 weeks), and old (50 weeks) WT and Thy1‐KO mice were studied. In the cortex of aged and old Thy1‐KO mice, the lack of THY1 is associated with an increased expression of the canonical astrocyte activation‐associated genes such as *Gfap, Vim*, *and Tnc* compared to WT mice (Figure 2A). No differences in the expression of these genes were observed in young animals, suggesting that the phenotype of Thy1‐KO mice develops with age. Therefore, mice aged 30–40 weeks were used in the further experiments. Next, astrocytes were isolated from the cortex using MACS to validate the astrocyte‐specific changes in gene expression in the absence of THY1. The single‐cell suspension obtained after tissue digestion contained about 40% ACSA‐2^+^ astrocytes, 30% CD11b^+^ microglial cells, and 4% oligodendrocytes (Figure S2A,B). Magnetic cell separation using anti‐ACSA‐2 microbeads revealed an astrocyte fraction of high purity (Figure S2C), which was used to investigate gene expression of a larger panel of activation‐associated markers in astrocytes isolated from the cortex of WT and Thy1‐KO mice. Similar to the data obtained from cortical tissue, deletion of *Thy1* increases the gene expression of the intermediate filaments *Gfap* and *Vim* in these purified astrocytes. In addition, the gene expression of *Kcnj10* and *Mki67* was increased, while other activation‐associated markers, including *S100b*, *Tgfb*, *Slc1a2*, *Slc1a3*, and *Cxcl10*, were not differentially expressed (Figure 2B). Consistent with these mRNA expression data, Thy1‐KO mice displayed a stronger immunofluorescence staining for GFAP in the cortex compared to WT mice (Figure 2C,D). Moreover, flow cytometry of single‐cell suspensions from the cortex showed a higher proportion of GFAP‐positive cells among the astrocytes detected by ACSA‐2 in Thy1‐KO than in WT mice (Figure 2E).

Taken together, the lack of Thy1 seems to support a distinct astrocyte phenotype characterized by increased expression of *Gfap*, *Vim*, *Kcnj10*, and *Mki67*, while other typical markers of reactive astrocytes are not affected by the loss of THY1.

### 3.3 Neuron‐Specific Deletion of THY1 Supports Astrocyte Activation

**FIGURE 3:**
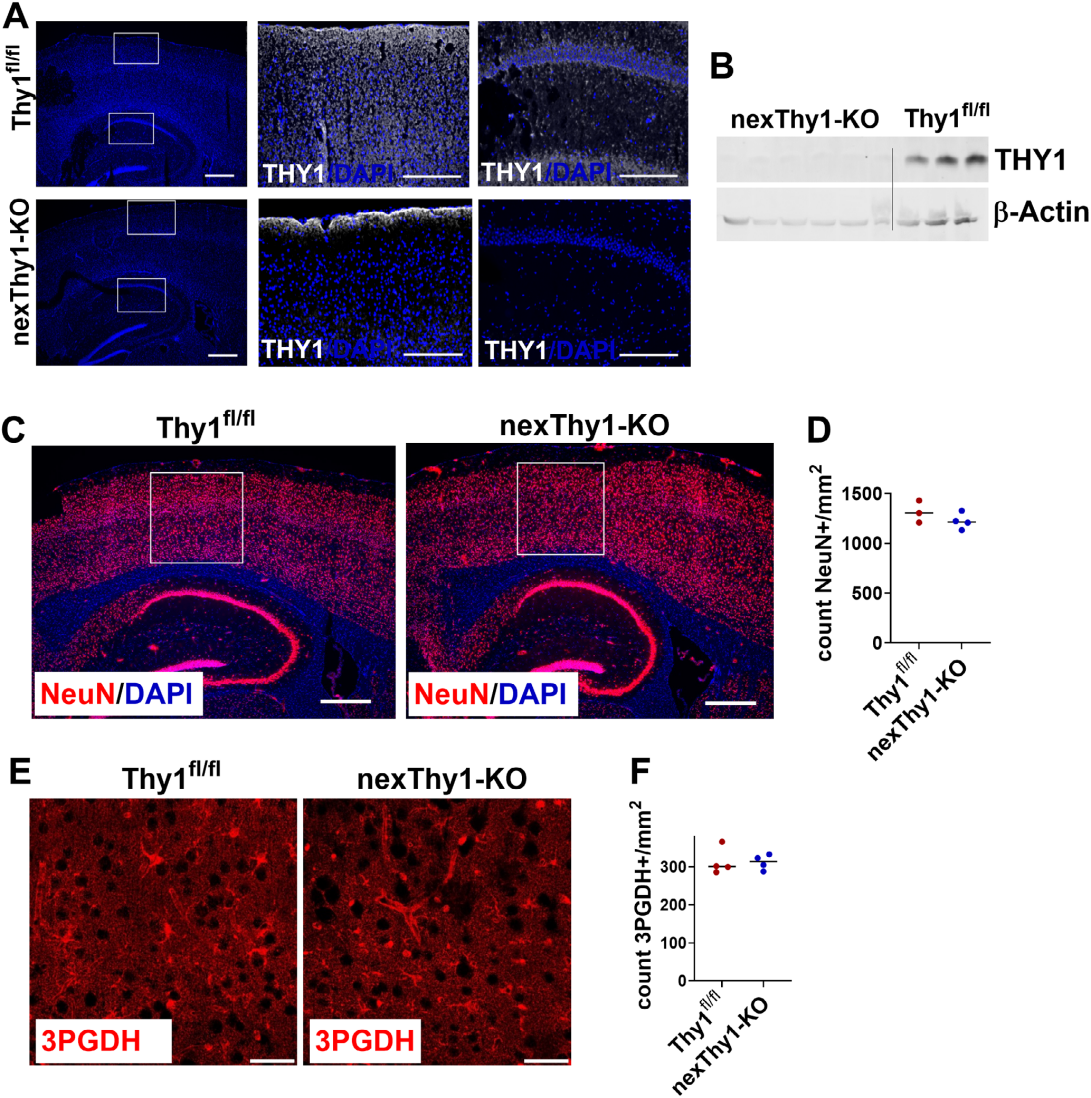
Characterization of mice with neuron‐specific *Thy1* deletion. Neuron‐specific knockout of the *Thy1* gene (nexThy1‐KO) was generated by crossing a mouse line with a loxP‐flanked *Thy1* allele to the Nex‐Cre driver line, which induces Cre‐dependent DNA recombination specifically in neurons of the forebrain. Mice were analyzed at an age of 30–40 weeks. (A) Immunofluorescence staining of THY1 (white) in brain slices from Thy1^fl/fl^ and nexThy1‐KO mice. Nuclei were labeled with DAPI (blue). White boxes indicate areas of higher magnification. Left panel: Scale bar: 500 μm, middle/right panel: Scale bar: 200 μm. (B) Detection of THY1 in tissue lysate from cortex of Thy1^fl/fl^ and nexThy1‐KO mice by Western blot. Anti‐β‐actin antibody was used as loading control. Representative examples are shown. (C) Detection of neurons in brain by NeuN (red) staining. One representative example per genotype is shown. Scale bar: 500 μm. (D) Number of NeuN^+^ cells was counted in the white box. (E) Astrocytes in the cortex were labeled by 3PGDH (red) staining. Scale bar: 50 μm. (F) The number of astrocytes was assessed by counting 3PGDH^+^ cells. The black line represents the mean. Each point represents one mouse (*n* = 3–4).

Since THY1 is expressed in various cell types (Saalbach and Anderegg 2019), we investigated whether the effects observed in whole‐body Thy1‐KO are related to neuronal THY1 expression using a neuron‐specific Thy1‐KO (nexThy1‐KO). While Thy1^flox/flox^ control mice display a strong THY1 expression in the cortex, *Thy1* gene and THY1 protein expression were only barely detectable in the cortex of nexThy1‐KO by immunofluorescence staining or Western Blot (Figure 3A,B). As observed in the whole‐body Thy1‐KO mice, neuron number and distribution were similar between nexThy1‐KO and control mice (Figure 3C,D). Moreover, the number of astrocytes detected by 3PGDH staining, a marker labeling almost all astrocytes in the cortex (Akdemir et al. 2020), was similar to that of control mice (Figure 3E,F). Finally, since deletion of the THY1‐receptor ITGB1 in astrocytes resulted in disturbed location of aquaporin 4 (AQP4) in astrocyte end feet (Robel et al. 2009), we analyzed AQP4 expression and distribution in nexThy1‐KO mice. Astrocyte endfeet, the main part of the blood–brain barrier, smoothly cover the endothelium of blood vessels in both Thy1^fl/fl^ and nexThy1‐KO mice (Figure S3).

**FIGURE 4:**
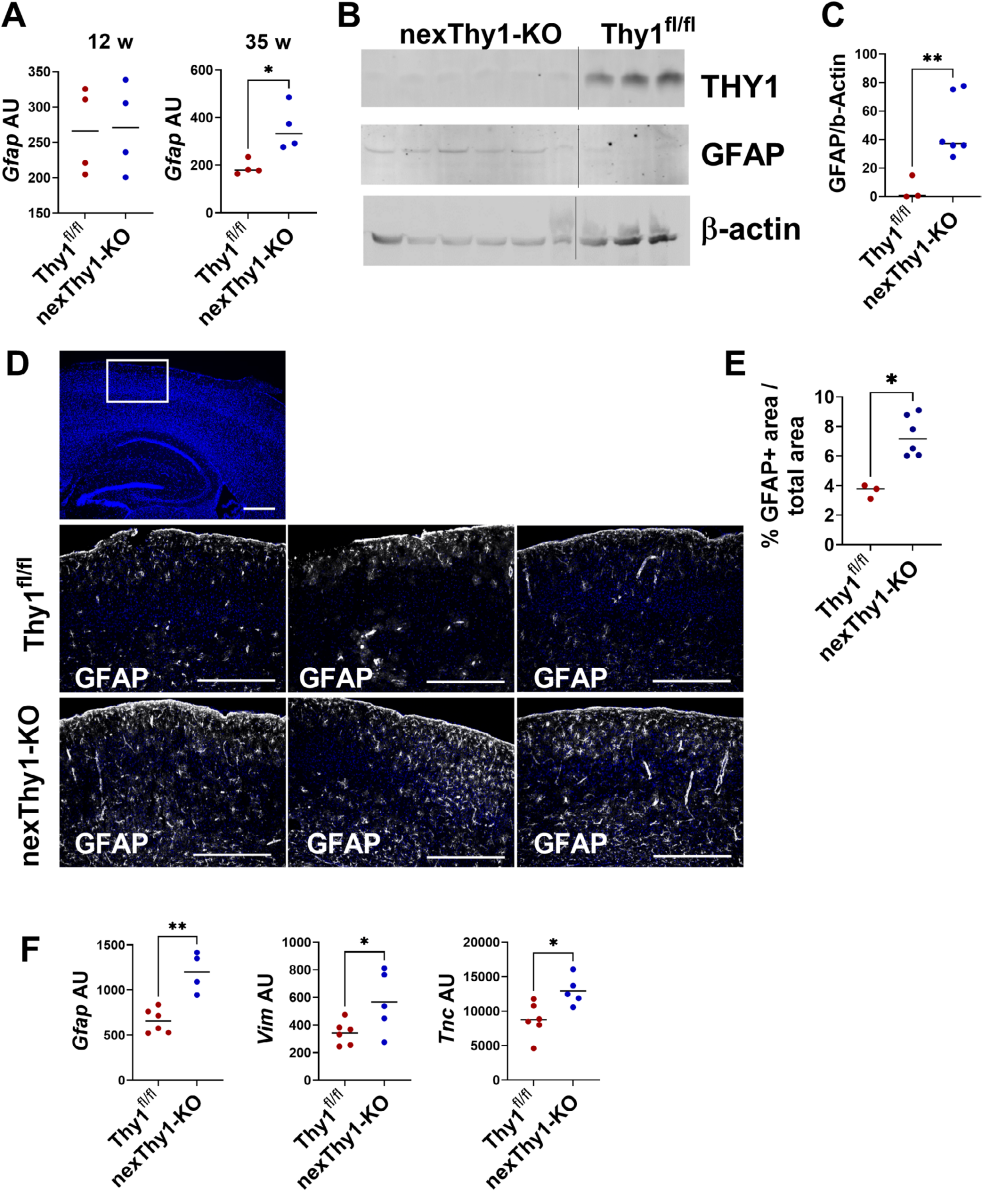
Neuron‐specific *Thy1* deletion induces upregulation of GFAP in astrocytes. GFAP expression was analyzed in the cortex of Thy1^fl/fl^ and nexThy1‐KO mice. (A) Gene expression detected by RT‐qPCR in 12 and 35 weeks (w) old Thy1^fl/fl^ and nexThy1‐KO mice. (B and C) Detection and quantification of GFAP in tissue lysate from the cortex of 35‐week‐old Thy1^fl/fl^ and nexThy1‐KO mice by Western blot. Anti‐β‐actin antibody was used as loading control. (B) Representative Western blot. (C) Quantitative analysis. (D and E) Detection of GFAP (white) in brain slices of 35‐week‐old Thy1^fl/fl^ and nexThy1‐KO mice by immunofluorescence staining. Nuclei were labeled with DAPI (blue). White box indicates the area of higher magnification. Three mice per genotype are shown (Scale bar: 500 μm upper panel; 200 μm lower panel). (E) Area of GFAP‐positive signal was evaluated. Percentage of GFAP+ area/total area is shown. (F) Astrocytes were isolated from 35‐week‐old Thy1^fl/fl^ and nexThy1‐KO mice by magnetic cell separation. Indicated genes were detected by RT‐qPCR. (A, C, E, and F) Each point represents one mouse (*n* = 4–6). The black line represents the mean. *p < 0.05, **p < 0.01, Student's *t*‐test.

**FIGURE 5:**
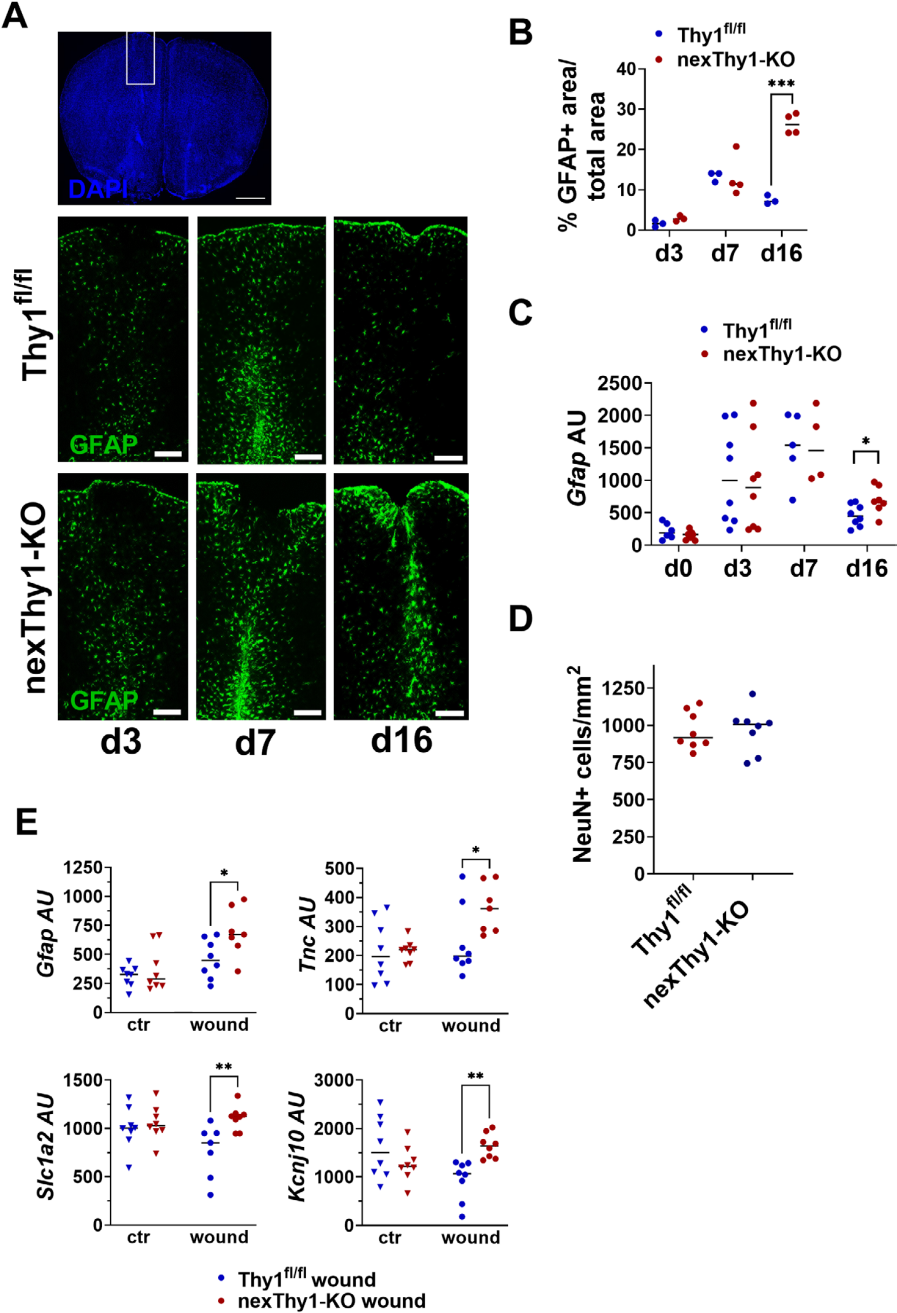
Neuron‐specific *Thy1* deletion prolongs astrocyte activation upon injury. Stab wounds were induced in 14‐week‐old Thy1^fl/fl^ and nexThy1‐KO mice. (A) GFAP staining (green) in representative tissue sections 3, 7, and 16 days after injury. White box indicates the area for quantification and magnification. Scale bar: 200 μm. (B) Area of GFAP‐positive signal was evaluated. Percentage of GFAP+ area/total area is shown. (C) *Gfap* gene expression analysis detected by RT‐qPCR. (D) The number of neurons in the lesion area was quantified using NeuN staining. (E) Gene expression analysis of indicated genes on the injured (wound) and contralateral, uninjured (ctr) side of the brain 16 days after lesion. Each point represents one mouse (*n* = 3–8). The black line represents the mean. *p < 0.05, **p < 0.01, ***p < 0.001, Student's *t*‐test.

Similar to the data from Thy1‐KO mice, gene expression analysis showed increased expression of *Gfap* in the cortex of nexThy1‐KO mice at the age of 35 weeks, while young mice (12 weeks) did not show any differences in *Gfap* expression (Figure 4A). Western Blot analysis (Figure 4B,C) and immunofluorescence staining of GFAP (Figure 4D,E) confirmed the increased GFAP protein expression in aged nexThy1‐KO compared to control mice. Analysis of MACS‐sorted astrocytes from control and nexThy1‐KO mice underlines the increased expression of *Gfap*, *Vim*, and *Tnc* in the absence of neuronal THY1 (Figure 4F). These data strongly indicate that, in particular, the neuronal expression of THY1 and, therefore, most likely direct THY1‐mediated signaling, is responsible for the suppression of astrocyte activation in aged mice. Since deletion of *Thy1* induces this partially activated astrocyte phenotype only in aged mice, but not in young adult mice, we hypothesized that THY1‐mediated regulation of astrocyte activation is especially relevant in challenging situations like aging or injury. Therefore, we induced stab wound lesions in young (14 weeks old) NexThy1‐KO and control mice. Quantification of *Gfap* gene and GFAP protein expression as a marker for astrocyte activation revealed injury‐induced astrocyte activation that peaked at day 7 and returned to basal level after 16 days in control mice (Figure 5A–C). In contrast, in the absence of neuronal THY1, a steadily rising astrocyte activation over time was prominent, which indicates an excessive response of cortical astrocytes and a delayed termination of their activation (Figure 5A–C). The number of neurons within the lesions was not different between control and Thy1‐KO mice (Figure 5D). Gene expression analysis underlines the prolonged activation of astrocytes upon stab injury in nexThy1‐KO mice. As shown in Figure 5E, expression of additional typical activation‐associated genes such as *Tnc*, *Kcnj10*, and *Slc1a2* is increased in the wounded tissue in the absence of neuronal THY1, but not on the contralateral, uninjured side.

### 3.4 THY1 Regulates Cell Death and Survival in Astrocytes

**FIGURE 6:**
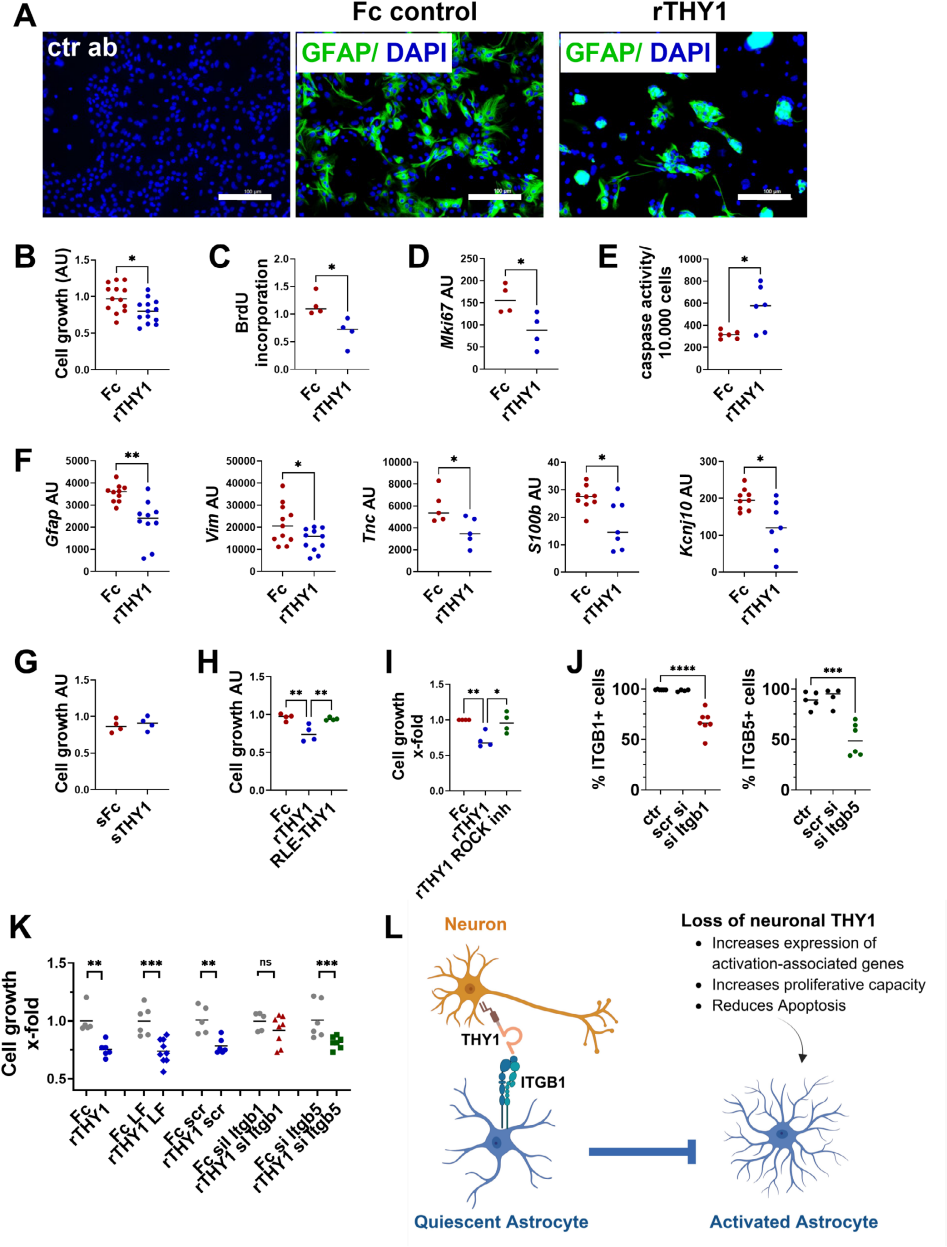
THY1 restricts the cell growth of astrocytes. Primary astrocytes were obtained from the cortex of wild‐type P0/1 mice. After 2 weeks in vitro, astrocytes were detached and cultured on immobilized recombinant THY1 (rTHY1) or control Fc protein (Fc) for 2 days. (A) Staining of GFAP in astrocytes (green) cultured on rTHY1 and Fc control protein. Nuclei were labeled by DAPI (blue). Isotype control antibody (ctr ab) was used as negative control. One representative example is shown. Scale bar: 100 μm. (B) XTT assay was used to evaluate cell growth. (C) Proliferation was quantified by BrdU incorporation. (D) *Mki67* gene expression detected by RT‐qPCR. (E) Detection of apoptosis by densitometric analysis of caspase activity per 10,000 cells. (F) Gene expression analysis of the indicated genes detected by RT‐qPCR. Cell growth was detected by XTT. (G) Astrocytes were incubated with soluble THY1 (sTHY1) or soluble control protein (sFc) for 2 days. Cell growth was detected by XTT. (H) Astrocytes were cultured on immobilized recombinant THY1 (rTHY1), control Fc protein (Fc), or mutated THY1 (RLE‐THY1) for 2 days. (I) Astrocytes were cultured on immobilized recombinant THY1 (rTHY1), control Fc protein (Fc) in the presence of 10 μM ROCK inhibitor for 2 days. X‐fold change in cell growth is shown. (J and K) TU2449 cell line was untreated (ctr), treated with lipofectamine (LF) alone, scrambled siRNA (scr), siRNA against *Itgb1* or *Itgb5*. (J) Detection of the percentage of ITGB1 and ITGB5 positive cells by flow cytometry. (K) Cell growth of these cells on plates coated with recombinant THY1 (rTHY1) or control protein (Fc). X‐fold change by THY1 coating compared to the Fc control protein is shown for each condition. (L) Schematic overview of THY1 action. (B–K) Each dot represents an experiment performed on an independent culture (*n* = 3–14). The black line represents the mean. *p < 0.05, **p < 0.01, ***p < 0.001, ****p < 0.0001, ns: non‐significant, Student's *t*‐test.

To test whether THY1 directly affects astrocytes, astrocytes were cultured in vitro on immobilized recombinant THY1 (rTHY1) or control protein (Fc). Astrocytes were isolated from the cortex of WT P0/P1 mice and cultured for 2 weeks. Flow cytometry analysis indicated a high purity of these astrocyte cultures (Figure S4). After 2 weeks, the cells were detached and cultured on rTHY1 or Fc for 2 days. Immobilized rTHY1 strongly affected the spreading of astrocytes (Figure 6A), inhibited cell growth as shown by XTT assay (Figure 6B) and reduced their proliferative capacity indicated by impaired BrdU incorporation (Figure 6C). Consistently, gene expression of the proliferation marker *Mki67* was lower upon contact of astrocytes with rTHY1 (Figure 6D). In addition, rTHY1 induced apoptosis of astrocytes as indicated by caspase activity (Figure 6E). Supporting the data from cortex and isolated astrocytes, the interaction of cultured astrocytes with rTHY1 reduced the expression of the intermediate filaments *Gfap*, *Tnc*, and *Vim*, as well as the expression of *S100b* and *Kcnj10* (Figure 6F). Interestingly, the addition of soluble THY1 (sTHY1) did not affect astrocyte cell growth (Figure 6G), suggesting that clustering/immobilization of THY1 is a prerequisite for effective signaling to astrocytes.

Next, the binding partners of Thy1 on astrocytes responsible for the THY1‐mediated control of astrocyte activation were identified. Several integrins have been described as binding partners for THY1. Indeed, when astrocytes were seeded on plates coated with mutated THY1 (RLD→RLE sequence), in which the integrin binding site is lost, astrocyte cell growth was not affected (Figure 6H). Moreover, inhibition of integrin signaling by Y27632, a selective small molecule inhibitor of Rho‐associated kinase (ROCK), reverses THY1‐mediated effects (Figure 6I). Our data indicate that THY1‐mediated control of astrocyte activation is mediated by binding to integrins on astrocytes. As shown before, astrocytes express ITGB1 and ITGB5, which are both described as THY1 receptors. To investigate which of these integrins mediates THY1‐induced signaling, their expression was downregulated by siRNA transfection in the astrocyte cell line TU2449, which was used instead of primary astrocytes to achieve high transfection efficiency. Transfection with siRNA against *Itgb1* and *Itgb5* significantly reduced protein expression of ITGB1 and ITGB5, respectively, as shown by flow cytometry (Figure 6J). Knockdown of ITGB1, but not of ITGB5, alleviated the cell growth inhibitory effect of THY1 (Figure 6K). Thus, THY1‐mediated control of astrocyte activation is mediated by binding to ITGB1 on astrocytes (Figure 6L).

Taken together, our in vitro and in vivo data demonstrate that the loss of neuronal THY1 fosters a distinct partially activated phenotype of astrocytes characterized by increased expression of a subset of activation‐associated genes, including intermediate filaments, enhanced proliferative capacity, and decreased cell death.

## 4 Discussion

THY1 is expressed on the surface of mature neurons and represents one of the most abundant cell‐surface glycoproteins in the nervous system (Saalbach and Anderegg 2019). The present study uncovered that neuronal THY1 is a still unrecognized novel regulator in the communication between astrocytes and neurons and thereby uniquely regulates the activation state of astrocytes. These findings add THY1 to the growing group of known cell surface molecules signaling between neurons and astrocytes. Further, the results provide evidence that downregulation of THY1, which is observed in brain injury (Chen et al. 2005) or in inflammatory conditions, enables the development of a specific activation state of astrocytes, which might be involved in the response to a fragile or disturbed condition of neurons, for instance, during brain injury or inflammation.

To investigate the impact of THY1 signaling on astrocytes, whole‐body Thy1‐KO mice, in which THY1 expression is lacking on all cells, including neurons (Morris 2018), as well as neuron‐specific conditional Thy1‐KO mice, were used. Counting of neurons by NeuN in the cortex of both mouse models did not indicate any general neurodegeneration, suggesting that the observed phenotype is a consequence of disturbed THY1 signaling rather than astrocytes' reaction to dying neurons. These findings are consistent with previous observations that Thy1‐KO mice display normal cellular organization, normal anatomical features of the corticospinal and thalamocortical pathways, and basic neurophysiological properties of thalamocortical synaptic transmission (Barlow et al. 2002).

When astrocytes are activated, they regulate genes controlling neurotransmitter uptake, ion homeostasis, water balance, exchange of molecules between neighboring cells, cytoskeletal stability, and synaptogenesis (Akdemir et al. 2020). Deletion of *Thy1* induced the gene expression of intermediate filaments, including *Gfap* and *Vim*, in astrocytes. Both are major protein constituents of astrocyte intermediate filaments, providing cytoskeletal stability, supporting synapse formation, motility, and adhesion (Akdemir et al. 2020). GFAP is a widely used marker of reactive astrocytes, and increased GFAP has been found in different CNS pathologies as an early response to injury (Escartin et al. 2021). In addition to GFAP, loss of THY1 signaling results in the upregulation of further markers of reactive astrocytes like *Mki67* and *Kcnj10*. Other typical markers of reactive astrocytes, such as *Slc1a2*, *Slc1a3*, *Tgfb*, and *S100b*, are not affected by loss of THY1 expression (Escartin et al. 2021). Comparison with datasets characterizing immature vs. mature astrocytes, reactive astrocytes, A1 vs. A2 astrocytes, or aged vs. young astrocytes (Cahoy et al. 2008; Akdemir et al. 2020; Fan and Huo 2021; Lattke et al. 2021; Lawrence et al. 2023) suggests that deletion of *Thy1* induces a specific phenotype of astrocyte reactivity characterized by a distinct set of molecular changes resulting in a “partially reactive” state. Consequently, THY1 might be responsible for the control of a distinct set of activation‐associated markers and functions. Consistently, deletion of the THY1‐receptor *Itgb1* in astrocytes induces a similar partially activated phenotype characterized by increased expression of *Gfap*, *Vim*, and *Tnc* (Robel et al. 2009). Astrocyte‐specific deletion of *Itgb1* results in mis‐localization of AQP4 at astrocyte endfeet and disturbed endfeet polarity (Robel et al. 2009). In contrast, mice with neuron‐specific deletion of *Thy1* do not display any alterations in the expression and localization of AQP4 at astrocyte endfeet, indicating that another ligand than neuronal THY1 is involved in the regulation of the morphology, polarity, and function of astrocytic endfeet.

Deletion of *Thy1* induced this partially activated astrocyte phenotype only in aged and old mice, but not in young adult mice, suggesting that under normal, young healthy conditions loss of THY1 expression is well tolerated and/or compensated by a so far unknown mechanism. Age‐related defects have also been observed in other knockout models like *Gfap* or *synuclein* KO mice, but the underlying mechanism is still unknown (Greten‐Harrison et al. 2010; Leipp et al. 2024). In contrast, under conditions of accumulating stress, such as during aging, THY1 seems to be important in the regulation of astroglia activation. Indeed, an extensive astrocytic response and the failure of its termination after brain injury provide further evidence for the role of THY1 in the control of astrocyte activation. Thus, THY1 seems to be an important player in the restoration of homeostatic conditions in the brain. In line, similar effects were observed in bleomycin‐induced lung fibrosis in *Thy1*‐deficient mice. These Thy1‐KO mice displayed persistent myofibroblast and collagen accumulation as well as decreased apoptosis of myofibroblasts, all resulting in a failure of lung fibrosis resolution (Liu et al. 2017).

Besides its appearance on neurons, THY1 is also expressed on other cell types, including fibroblasts, microvascular endothelial cells, mesenchymal stem cells, and—in mice—T cells under healthy conditions (Saalbach and Anderegg 2019). In the CNS, fibroblasts are mostly found in the meninges, perivascular Virchow‐Robin space, and choroid plexus (Duan and Yu 2024). Recent work revealed that fibroblasts play crucial roles in fibrotic scar formation in the CNS after injury and inflammation (Dorrier et al. 2022). Furthermore, astrocytes are in contact with endothelial cells with their end feet, while invading T cells might also affect astrocytes' activation state. To show that the phenotype observed in Thy1‐KO mice is due to a loss of THY1 expression on neurons, we generated a mouse line with neuron‐specific knockout of the *Thy1* gene (nexThy1‐KO) in which THY1 expression is lost specifically in neurons of the forebrain, but not in other cell types expressing THY1, such as fibroblasts or T cells. Immunofluorescence staining and Western blot analysis showed the successful deletion of *Thy1* in neurons in the brain of these mice. The number of astrocytes is not affected by neuron‐specific deletion of *Thy1*. However, as shown in the whole‐body Thy1‐KO, astrocytes from neuron‐specific Thy1‐KO mice display a partially activated phenotype, providing evidence that this phenotype is caused by the loss of direct signaling of neuronal THY1 to astrocytes. Vice versa, these findings suggest that under physiological conditions, that is, in the presence of THY1, neurons inhibit astrocytes' reactivity, thereby keeping them in a physiological, nonreactive state. During pathological events, neurons downregulate THY1 (Chen et al. 2005), thereby releasing this handbrake to allow astrocytes to react to the pathological insult by partial reactive astrogliosis and, most likely, contribute to mitigate the injury.

To provide further evidence of direct signaling of THY1 to astrocytes, primary cortical astrocytes were cultured on tissue culture plates coated with recombinant THY1. Indeed, the presence of THY1 inhibited the expression of *Gfap*, *Vim*, *S100b*, *Slc1a2*, and *Kcnj10*, showing that THY1‐mediated signaling directly controls the expression of these genes associated with astrocyte reactivity. Furthermore, functional assays demonstrate that the presence of THY1 restricts cell growth and proliferation, while it promotes apoptosis of astrocytes. Importantly, immobilization and/or crosslinking of THY1 seems to be important since soluble THY1 does not affect astrocyte activation. Consistently, the control of cell growth and apoptosis by THY1 has been shown in other cell types. Lack of THY1 on dermal fibroblasts results in decreased apoptosis and a higher proliferation rate (Schmidt et al. 2016). Mechanistic studies in fibroblasts demonstrated that THY1 binding to ITGB3 stimulates the expression of Fas‐ligand, which in turn activates a pro‐apoptotic caspase cascade (Schmidt et al. 2016). Consistently, mice lacking THY1 develop more severe lung, heart, liver, and kidney fibrosis than WT controls (Hagood et al. 2005; Li et al. 2020; Nishio et al. 2021; Blank et al. 2024). In addition, similar to astrocytes, THY1 restricts proliferation while promoting apoptosis of non‐parenchymal cells isolated from the liver (Blank et al. 2024).

Several studies suggest that THY1 interaction with integrins is important in the functional network of neurons and astrocytes. Using an astrocyte cell line, it has been described that the interaction of neuronal THY1 with ITGB3 on astrocytes launches signaling cascades in both cell types, regulating cell attachment, cytoskeleton organization, spreading, and migration of astrocytes as well as the intracellular Ca^2+^ in astrocytes (Leyton et al. 2001, 2019; Hermosilla et al. 2008; Avalos et al. 2009). However, the relevance of these findings for astrocytes in vivo is unclear, as we could not detect ITGB3 on astrocytes, consistent with expression data from cell type‐specific expression databases (Zhang et al. 2014). In contrast, astrocytes express ITGB1 and ITGB5, two integrins known to interact with THY1 (Zhou et al. 2010; Fiore et al. 2014). Both integrins are able to bind THY1 in cis. At the single‐molecule level, THY1 is capable of binding α5β1 integrin and Syn4 receptors, resulting in the formation of a trimolecular complex (Fiore et al. 2014). The interaction of THY1 with ITGB5 inhibits contraction‐induced latent TGF‐β1 activation of myofibroblasts (Zhou et al. 2010). Until now, there are no data about the role of these integrins in the bidirectional communication between astrocytes and neurons. The failure of THY1‐mediated control of astrocyte activation by THY1 without an integrin binding site, the reversal of THY1‐mediated effects upon blocking integrin signaling, as well as the knockdown of ITGB1 but not ITGB5, indicates that THY1 controls astrocyte activation via the interaction with ITGB1 on astrocytes.

In summary, the interaction of neuronal THY1 with ITGB1 on astrocytes contributes to keeping astrocytes in a quiescent, nonreactive state. Loss of THY1 allows the activation of astrocytes and results in the development of a distinct, partially activated astrocyte phenotype characterized by increased expression of intermediate filaments, increased proliferative capacity, and reduced cell death. Moreover, downregulation of ITGB1 in inflammatory conditions may contribute to the release of the THY1‐mediated handbrake of astrocyte activation, allowing full astrocyte response to inflammation and injury. Thus, our data suggest that THY1‐mediated neuron‐to‐astrocyte signaling is an important mechanism in the maintenance and restoration of brain tissue homeostasis.

## Author Contributions

A.S. designed the study, performed experiments, analyzed data and wrote the manuscript. J.L. performed experiments, analyzed data and edited the manuscript. D.J. provided methods, edited the manuscript. J.H. discussed study design and data, provided methods, edited the manuscript. A.R. and U.W. performed experiments and edited the manuscript. All authors discussed the data, read the manuscript.

## Ethics Statement

All animal experiments were performed in accordance with institutional and state guidelines and were approved by the Committee on Animal Welfare of Saxony, Germany (TVV39/23, TVV26/19, and T05/21‐MEZ).

## Conflicts of Interest

The authors declare no conflicts of interest.

## Supporting information


**Figure S1:** Characterization of Thy1‐KO mice. (A) Detection of THY1 (white) in brain slices from 12‐ to 14‐week‐old wild‐type (WT) and total Thy1‐KO mice by immunofluorescence staining. Nuclei were labeled with DAPI (blue). Scale bar: 500 μm. (B) Detection of THY1 in tissue lysate from cortex of WT and Thy1‐KO mice by Western blot. Anti‐RPL26 was used as loading control. Representative examples are shown. (C) Detection of neurons in brain labeled by NeuN (red). One representative example is shown. Scale bar: 500 μm. (D) Quantification of NeuN^+^ neurons in the area indicated by the white box in (C). Each dot represents one mouse (*n* = 3 mice). Mean is shown. (E) Gating strategy of flow cytometry analysis of the cortex of WT and Thy1‐KO mice. Dead cells were excluded by Zombie staining. Oligodendrocytes were detected by O4 staining. O4^−^ cells were analyzed for astrocytes (ACSA‐2^+^ cells) and microglial cells (CD11b^+^ cells). Neurons were identified by negative staining for ACSA‐2, CD11b, and O4. One representative experiment of three is shown. (F) Percentage of ACSA‐2^+^ (astrocytes, AC), CD11b^+^ (microglia, MG), O4^+^ (oligodendrocytes, OG), and CD11b^−^/ACSA‐2^−^/O4^−^ (neurons, Neu) was detected in WT and Thy1‐KO mice (*n* = 3). Each point represents one mouse (*n* = 3). The black line represents the mean.
**Figure S2:** Purity of the astrocyte cell population obtained by MACS from mice at the age of 30–40 weeks. Cortex was enzymatically digested, and astrocytes were isolated by magnetic cell separation using ACSA‐2 beads. Purity was checked by flow cytometry analysis. (A) Gating strategy. (B and C) Detection of astrocytes (ACSA‐2^+^), oligodendrocytes (O4^+^), and microglial cells (CD11b^+^). (B) before and (C) after separation. One representative example of three is shown.
**Figure S3:** AQP4 and 3PGDH expression. Astrocytes in the cortex of Thy1^fl/fl^ and nexThy1‐KO mice were co‐stained with the astrocyte marker 3PGDH (red) and AQP4 (turquoise). Scale bar: 10 μm.
**Figure S4:** Purity of primary astrocyte cultures. Astrocytes were generated from the cortex of wild‐type P0/P1 mice. After 2 weeks, astrocytes were detached and purity was checked by flow cytometry analysis. Detection of astrocytes (93% ACSA‐2^+^), oligodendrocytes (2% O4^+^), and microglial cells (5% CD11b^+^). One representative example of three is shown.
**Table S1:** Sequences of primers.
**Table S2:** Antibodies used in this study.

## Data Availability

The data that support the findings of this study are available in Supporting Information S1 of this article.
