# Supplementary material for: Neuronal THY1 Signaling Maintains Astrocytes in a Quiescent State
Source: Glia. 2025 Sep 11;74(1):e70083. doi: 10.1002/glia.70083 (PMC12666997; doi:10.1002/glia.70083)
Supplement: Supplementary file 1 — Figure S1: Characterization of Thy1‐KO mice. (A) Detection of THY1 (white) in brain slices from 12‐ to 14‐week‐old wild‐type (WT) and total Thy1‐KO mice by immunofluorescence staining. Nuclei were labeled with DAPI (blue). Scale bar: 500 μm. (B) Detection of THY1 in tissue lysate from cortex of WT and Thy1‐KO mice by Western blot. Anti‐RPL26 was used as loading control. Representative examples are shown. (C) Detection of neurons in brain labeled by NeuN (red). One representative example is shown. Scale bar: 500 μm. (D) Quantification of NeuN+ neurons in the area indicated by the white box in (C). Each dot represents one mouse (n = 3 mice). Mean is shown. (E) Gating strategy of flow cytometry analysis of the cortex of WT and Thy1‐KO mice. Dead cells were excluded by Zombie staining. Oligodendrocytes were detected by O4 staining. O4− cells were analyzed for astrocytes (ACSA‐2+ cells) and microglial cells (CD11b+ cells). Neurons were identified by negative staining for ACSA‐2, CD11b, and O4. One representative experiment of three is shown. (F) Percentage of ACSA‐2+ (astrocytes, AC), CD11b+ (microglia, MG), O4+ (oligodendrocytes, OG), and CD11b−/ACSA‐2−/O4− (neurons, Neu) was detected in WT and Thy1‐KO mice (n = 3). Each point represents one mouse (n = 3). The black line represents the mean. Figure S2: Purity of the astrocyte cell population obtained by MACS from mice at the age of 30–40 weeks. Cortex was enzymatically digested, and astrocytes were isolated by magnetic cell separation using ACSA‐2 beads. Purity was checked by flow cytometry analysis. (A) Gating strategy. (B and C) Detection of astrocytes (ACSA‐2+), oligodendrocytes (O4+), and microglial cells (CD11b+). (B) before and (C) after separation. One representative example of three is shown. Figure S3: AQP4 and 3PGDH expression. Astrocytes in the cortex of Thy1fl/fl and nexThy1‐KO mice were co‐stained with the astrocyte marker 3PGDH (red) and AQP4 (turquoise). Scale bar: 10 μm. Figure S4: Purity of primary astr [file GLIA-74-0-s001.docx]

**Supplementary Figures**

**
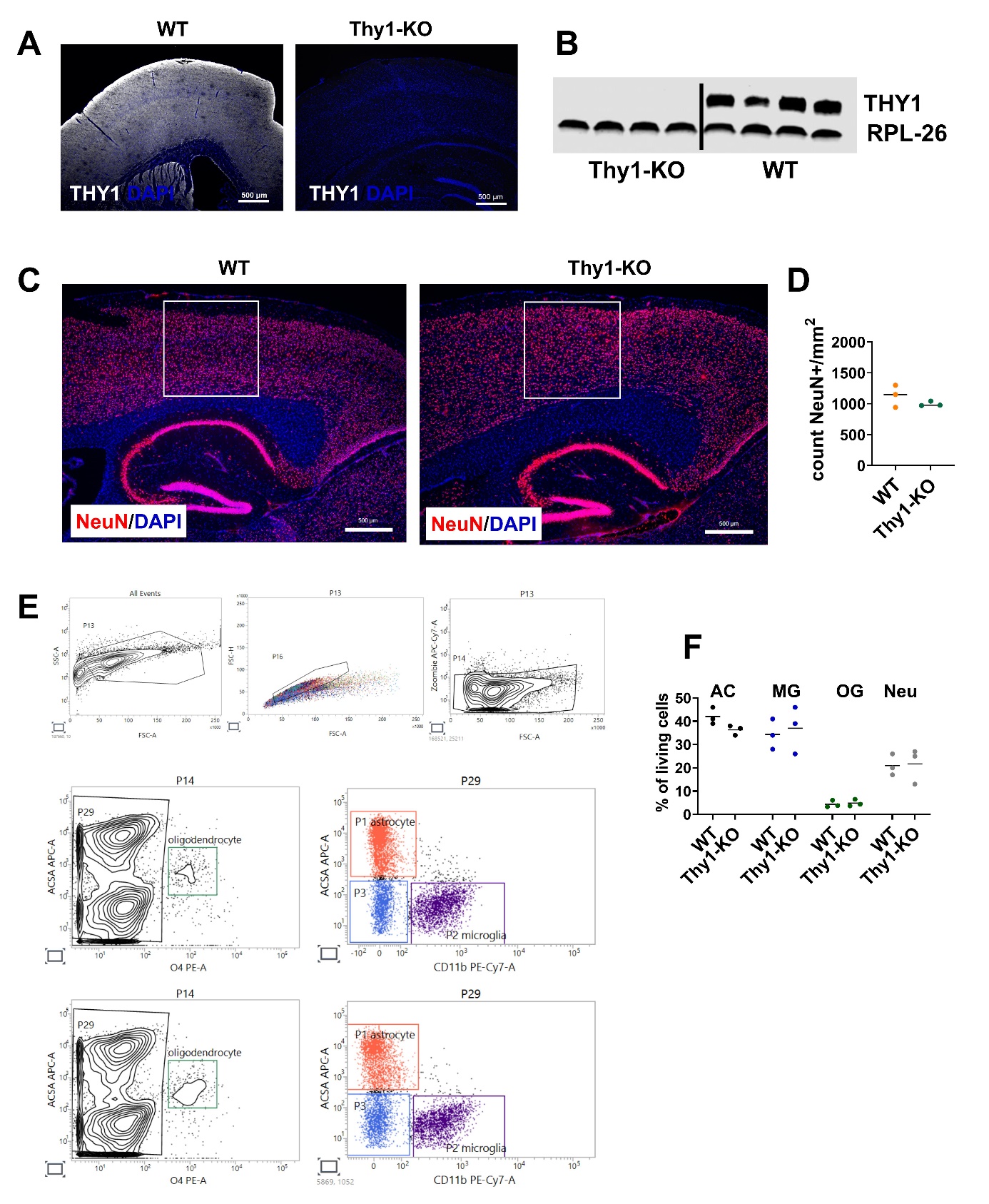
**

**Supplementary Figure 1: Characterisation of Thy1-KO mice**

1. Detection of THY1 (white) in brain slices from 12- to 14-week-old wild-type (WT) and total Thy1-KO mice by immunofluorescence staining. Nuclei were labelled with DAPI (blue). Scale Bar: 500 µm. **B)** Detection of THY1 in tissue lysate from cortex of WT and Thy1-KO mice by Western Blot. Anti-RPL26 was used as loading control. Representative examples are shown. **C)** Detection of neurons in brain labelled by NeuN (red). One representative example is shown. Scale Bar: 500 µm. **D)** Quantification of NeuN+ neurons in the area indicated by the white box in (C). Each dot represents one mouse (n=3 mice). Mean is shown. **E)** Gating strategy of flow cytometry analysis of the cortex of WT and Thy1-KO mice. Dead cells were excluded by Zombie staining. Oligodendrocytes were detected by O4 staining. O4^-^ cells were analysed for astrocytes (ACSA-2^+^ cells) and microglial cells (CD11b+ cells). Neurons were identified by negative staining for ACSA-2, CD11b and O4. One representative experiment of three is shown. **F)** Percentage of ACSA-2+ (astrocytes, AC), CD11b+ (microglia, MG), O4+ (oligodendrocyte, OG), and CD11b-/ACSA-2-/O4- (neurons, Neu) was detected in WT and Thy1-KO mice (n=3). Each point represents one mouse (n=3). The black line represents the mean.


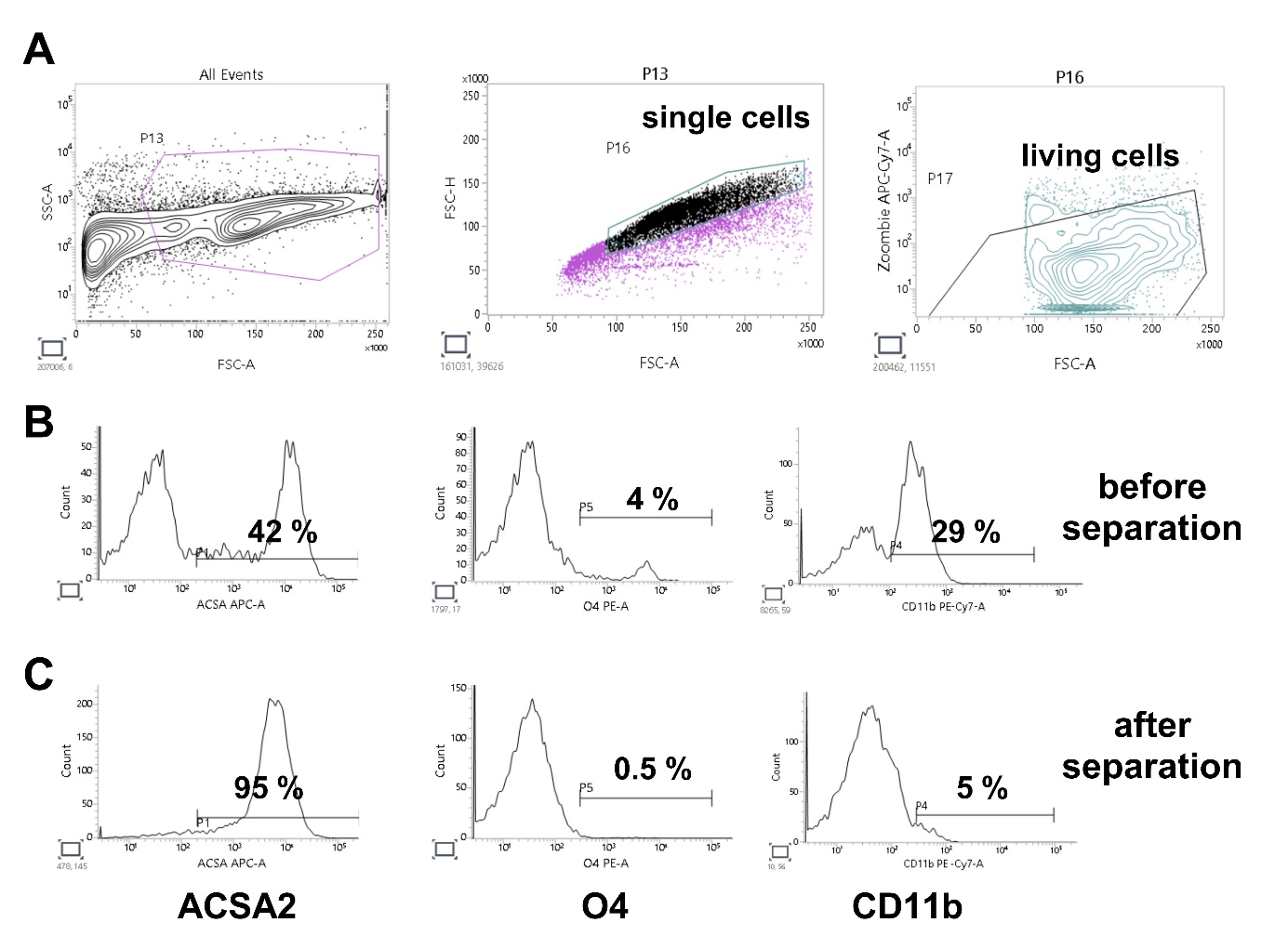


**Supplementary Figure 2: Purity of the astrocyte cell population obtained by MACS from mice at the age of 30-40 weeks.**

Cortex was enzymatically digested and astrocytes was isolated by magnetic cell separation using ACSA-2 beads. Purity was checked by flow cytometry analysis. **A)** Gating strategy. **B/C)** Detection of astrocytes (ACSA-2+), oligodendrocytes (O4+) and microglial cells (CD11b+). B) before and C) after separation. One representative example of three is shown.

**
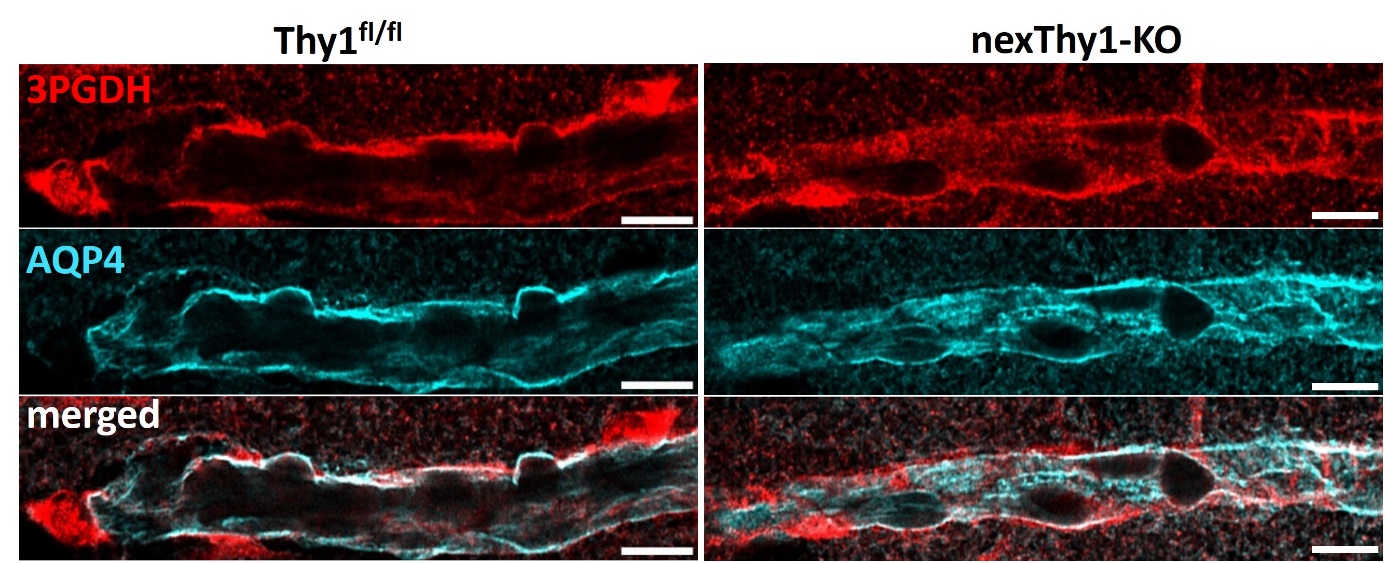
**

**Supplementary Figure 3: AQP4 and 3PGDH expression**

Astrocytes in the cortex of Thy1^fl/fl^ and nexThy1-KO mice were co-stained with the astrocyte marker 3PGDH (red) and AQP4 (turquoise). Scale bar: 10 µm

**
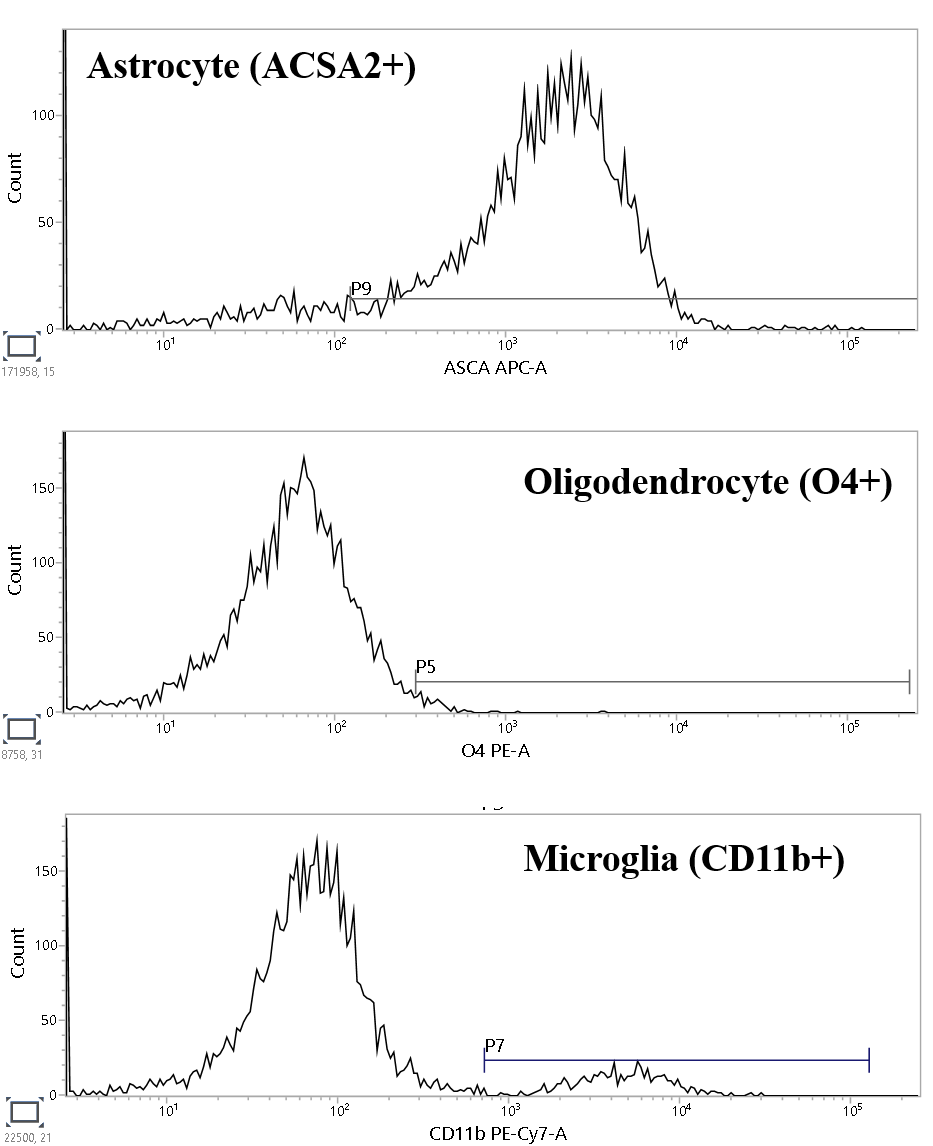
**

**Supplementary Figure 4: Purity of primary astrocyte cultures**

Astrocytes were generated from the cortex of wild-type P0/P1 mice. After two weeks astrocytes were detached and purity was checked by flow cytometry analysis. Detection of astrocytes (93 % ACSA-2+), oligodendrocytes (2 % O4+) and microglial cells (5 % CD11b+). One representative example of three is shown.

**SupplTab. 1: Sequences of primers**

| Gene | Primer sequence | Accession |
| --- | --- | --- |
| *Gfap*  Forward  Reverse | CTGGAGGGCGAAGAAAACCG  CTGGTGAGCCTGTATTGGGA | NM_001131020.1 |
| *Vimentin*  Forward  Reverse | GCAGTATGAAAGCGTGGCTG  CTCCAGGGACTCGTTAGTGC | NM_011701.4 |
| *Tenascin C*  Forward  Reverse | AACAACAGCCATGGGTTCTC  ACCCGGGGTAAGCTTCAC | NM_011607.3 |
| *S100b*  Forward  Reverse | CGGACACTGAAGCCAGAGAG  CCATCCCCATCTTCGTCCAG | NM_009115.3 |
| *Mki67*  Forward  Reverse | GATGCAAAAACTCTGAAGGAGG  GGAGGTGAAAACCACACTGG | NM_001081117.2 |
| *Kcnj10*  Forward  Reverse | GAGGGTCCTCACGAAAGACG  ACCACACCACACCAAAGAGG | NM_001039484.1 |
| *Slc1a2*  Forward  Reverse | GGATATCAGTCTGCTGGTGGC  CATTGGCCGCCAGAGTTACC | NM_001077514.4 |
| *Slc1a3*  Forward  Reverse | GTGCTTCGGTTTCGTGATCG  CAGGGGTTCTTCCGGGTTAC | NM_148938.3 |
| *Cxcl10*  Forward  Reverse | GTCATTTTCTGCCTCATCCTGC  GCTTCACTCCAGTTAAGGAGC | NM_021274.2 |
| *Tgfb*  Forward  Reverse | CTTCAATACGTCAGACATTCG  GTCAAAAGACAGCCACTCAG | NM_011577 |
| *Rplp0*  Forward  Reverse | GGACCCGAGAAGACCTCCTT  GCACATCACTCAGAATTTCAATGG | NM_007475 |

**SupplTab. 2: Antibodies used in this study**

| **Antibody against** | **Company** | **Order number** | **Clone** | **Host** | **Reactivity** | **Application** |
| --- | --- | --- | --- | --- | --- | --- |
| **Primary antibody** | | | | | | |
| THY1 | BD Pharmingen | 553016 | Clone G7 | rat | mouse | WB: 1 µg/ml  IF: 2µg/ml |
| GFAP | Cell Signaling | 12389 | Clone D1F4Q | rabbit | mouse | IF: 1:200  WB: 1:1000 |
| NeuN | Chemicon/Millipore | MAB377 | A60 | mouse | mouse | 1 mg/ml  IF: 1:100 |
| RPL26 | Sigma-Aldrich | R0655 | Polyclonal | rabbit | mouse | WB: 1 µg/ml |
| Beta-actin | Cell Signaling | 4967 | Polyclonal | rabbit | mouse | WB: 1:1000 |
| O4-PE | Miltenyi | 130-117-357 | Clone O4 | mouse | mouse | FC: 1:100 |
| ACSA-2-APC | Miltenyi | 130-116-245 | REA969 | recombinant antibody | mouse | FC: 1:100 |
| Thy1-PE | Miltenyi | 130-120-897 | REA1167 | recombinant antibody | mouse | FC: 1:100 |
| CD11b-PECY7 | Biolegend | 101216 | M1/70 | rat | mouse | FC: 1:100 |
| CD29 PE | Miltenyi | 130-102-994 | HMb1 | hamster | mouse | FC: 1:100 |
| CD61-PE | Miltenyi | 130-102-628 | 2C9.G2 | hamster | mouse/rat | FC: 1:100 |
| ITGB5 | R&D | AF8035_SP | polyclonal | sheep | mouse/rat | FC:1µg/ml |
| 3PGDH | Frontier Institute |  |  |  |  | IF: 1:200 |
| Aquaporin 4 (AQP4) | Sigma-Aldrich | A5971 | polyclonal | rabbit | Human/ mouse/rat | IF: 1:200 |
| **Secondary antibody** | | | | | | |
|  | Jackson ImmunoResearch |  |  | goat | Guinea pig | IF: 1:500 |
|  | Jackson ImmunoResearch |  |  | goat | Rabbit | IF: 1:500 |
|  | Invitrogen, Massachusetts, USA |  |  | goat | Rat | IF: 1:500 |
|  | Invitrogen, Massachusetts, USA |  |  | goat | Rabbit | IF: 1:500 |

IF: immunofluorescence staining, WB: western blot, FC: flow cytometry
